# Supplementary material for: Blebbisomes are large, organelle-rich extracellular vesicles with cell-like properties
Source: Nat Cell Biol. 2025 Feb 21;27(3):438–48. doi: 10.1038/s41556-025-01621-0 (PMC11906356; doi:10.1038/s41556-025-01621-0)
Supplement: Supplementary file 1 — Reporting Summary [file 41556_2025_1621_MOESM1_ESM.pdf]

nature portfolio

Corresponding author(s): Dennis K. Jeppesen
Dylan T. Burnette

Last updated by author(s): Sep 26, 2023

Reporting Summary

Nature Portfolio wishes to improve the reproducibility of the work that we publish. This form provides structure for consistency and transparency in reporting. For further information on Nature Portfolio policies, see our Editorial Policies and the Editorial Policy Checklist.

Statistics

For all statistical analyses, confirm that the following items are present in the figure legend, table legend, main text, or Methods section.

n/a	Confirmed	
☐	☒	The exact sample size (*n*) for each experimental group/condition, given as a discrete number and unit of measurement
☐	☒	A statement on whether measurements were taken from distinct samples or whether the same sample was measured repeatedly
☐	☒	The statistical test(s) used AND whether they are one- or two-sided<br>*Only common tests should be described solely by name; describe more complex techniques in the Methods section.*
☐	☒	A description of all covariates tested
☐	☒	A description of any assumptions or corrections, such as tests of normality and adjustment for multiple comparisons
☐	☒	A full description of the statistical parameters including central tendency (e.g. means) or other basic estimates (e.g. regression coefficient) AND variation (e.g. standard deviation) or associated estimates of uncertainty (e.g. confidence intervals)
☐	☒	For null hypothesis testing, the test statistic (e.g. *F*, *t*, *r*) with confidence intervals, effect sizes, degrees of freedom and *P* value noted<br>*Give P values as exact values whenever suitable.*
☒	☐	For Bayesian analysis, information on the choice of priors and Markov chain Monte Carlo settings
☒	☐	For hierarchical and complex designs, identification of the appropriate level for tests and full reporting of outcomes
☒	☐	Estimates of effect sizes (e.g. Cohen's *d*, Pearson's *r*), indicating how they were calculated

Our web collection on statistics for biologists contains articles on many of the points above.

Software and code

Policy information about availability of computer code

Data collection	VisiView Version 5.0.0.27 (Visitron Systems) was used to acquire all iSIM data.
Data analysis	FIJI, PRISM, and SuperPlots

For manuscripts utilizing custom algorithms or software that are central to the research but not yet described in published literature, software must be made available to editors and reviewers. We strongly encourage code deposition in a community repository (e.g. GitHub). See the Nature Portfolio guidelines for submitting code & software for further information.

Data

Policy information about availability of data

All manuscripts must include a data availability statement. This statement should provide the following information, where applicable:

- Accession codes, unique identifiers, or web links for publicly available datasets
- A description of any restrictions on data availability
- For clinical datasets or third party data, please ensure that the statement adheres to our policy

Mass spectrometry data have been deposited in ProteomeXchange with the primary accession code PXD059407 https://proteomecentral.proteomexchange.org/cgi/GetDataset?ID=PXD059407. All other data supporting the findings of this study are available from the corresponding author on reasonable request.

Research involving human participants, their data, or biological material

Policy information about studies with human participants or human data. See also policy information about sex, gender (identity/presentation), and sexual orientation and race, ethnicity and racism.

Reporting on sex and gender	N/A
Reporting on race, ethnicity, or other socially relevant groupings	N/A
Population characteristics	N/A
Recruitment	N/A
Ethics oversight	N/A

Note that full information on the approval of the study protocol must also be provided in the manuscript.

Field-specific reporting

Please select the one below that is the best fit for your research. If you are not sure, read the appropriate sections before making your selection.

☒ Life sciences ☐ Behavioural & social sciences ☐ Ecological, evolutionary & environmental sciences

For a reference copy of the document with all sections, see nature.com/documents/nr-reporting-summary-flat.pdf

Life sciences study design

All studies must disclose on these points even when the disclosure is negative.

Sample size	Sample sizes for experiments involving individual blebbisome measurements were not specifically calculated. Instead, every blebbisome in each data set was counted and assessed based on the parameters of the experiment. For the TMRE data, the sample size was chosen to be one because of the limitations of live cell imaging as well as likelihood of finding multiple blebbisomes in the same field of view as a cell for comparison.
Data exclusions	Data points were not excluded.
Replication	Each experiment has at least three biological replicates to provide adequate data and account for variability in sample preparation. Each attempt at replication was successful.
Randomization	Randomization is not relevant to this study, for the experiments were not designed to be compared to different treatment groups that would require randomization to eliminate bias.
Blinding	Blinding was not possible for these data sets due to the inherent design of the experiments in which microscopy images were taken specifically of blebbisomes and which were then assessed for characteristics of interest. The regions of the sample that were imaged were chosen blindly and blebbisomes were identified after acquisition.

Reporting for specific materials, systems and methods

We require information from authors about some types of materials, experimental systems and methods used in many studies. Here, indicate whether each material, system or method listed is relevant to your study. If you are not sure if a list item applies to your research, read the appropriate section before selecting a response.

Materials & experimental systems

n/a	Involved in the study	
☐	☒	Antibodies
☐	☒	Eukaryotic cell lines
☒	☐	Palaeontology and archaeology
☐	☒	Animals and other organisms
☒	☐	Clinical data
☒	☐	Dual use research of concern
☒	☐	Plants

Methods

n/a	Involved in the study	
☒	☐	ChIP-seq
☒	☐	Flow cytometry
☒	☐	MRI-based neuroimaging

Antibodies

Antibodies used

Monoclonal Mouse anti-GM130 BD Biosciences 610822
Polyclonal Rabbit anti-NMIIA BioLegend PRB-440P
Monoclonal Rabbit anti-RPS10 Abcam ab151550
Monoclonal Mouse anti-Mitochondria Abcam ab92824
Monoclonal Rabbit anti-HSP60 Cell Signaling Technology 12165
Monoclonal Rabbit anti-Annexin A1 Abcam ab214486
Monoclonal Rabbit anti-Annexin A2 Abcam ab178677
Monoclonal Rabbit anti-Syntenin Abcam ab133267
Polyclonal Rabbit anti-VDAC2 Abcam ab155803
Monoclonal Rabbit anti-NMIIA Abcam ab138498
Monoclonal Rabbity anti-RPS8 Abcam ab201454
Monoclonal Rabbit anti-Lamin A/C Abcam ab108595
Monoclonal Rabbit anti-Lamin A/C Abcam ab169532
Monoclonal Rabbit anti-EEF2 Abcam ab75748
Monoclonal Rabbit anti-Calreticulin Abcam Ab92516
Monoclonal Rabbit anti- Alpha Tubulin Abcam Ab52866
Monoclonal Rabbit anti- Cytokeratin 19 Abcam Ab52625
Monoclonal Rabbit anti-LC3B Abcam Ab192890
Monoclonal Rabbit anti-SQSTM1 / p62 Abcam Ab109012
Monoclonal Rabbit anti-LAMP1 Abcam Ab108597
Monoclonal Rabbit anti-LAMP1 Abcam Ab208943
Monoclonal Rabbit anti-LAMP2 Abcam Ab199946
Monoclonal Rabbit anti-GLUT1 Abcam Ab115730
Monoclonal Rabbit anti-TSG101 Abcam Ab125011
Monoclonal Rabbit anti-Alpha Actinin 4 Abcam Ab108198
Monoclonal Rabbit anti-CD63 Abcam Ab217345
Monoclonal Rabbit anti-Flotillin 1 Abcam Ab133497
Monoclonal Rabbit anti-RAB13 Abcam Ab205528
Monoclonal Rabbit anti-CD147 Abcam Ab108308
Monoclonal Rabbit anti-CD147 Abcam Ab188190
Monoclonal Mouse anti-HLA E Abcam Ab2216
Monoclonal Rabbit anti-CD47 Abcam Ab300124
Monoclonal Rabbit anti-Nectin 2 Abcam Ab135246
Monoclonal Rabbit anti-CD73 Abcam Ab133582
Monoclonal Rabbit anti-VISTA Abcam Ab300042
Monoclonal Rabbit anti-PVR Abcam Ab205304
Monoclonal Rabbit anti-PVR Abcam Ab267788
Monoclonal Rabbit anti-B7H4 Abcam Ab252438
Monoclonal Rabbit anti-CD276 Abcam Ab219648
Monoclonal Rabbit anti-CD276 Abcam Ab134161
Monoclonal Rabbit anti-PD-L2 Abcam Ab288298
Monoclonal Rabbit anti-PD-L2 Abcam Ab256386
Monoclonal Rabbit anti-PD-L1 Abcam Ab213480
Monoclonal Rabbit anti-PD-L1 Abcam Ab213524
Monoclonal Rabbit anti-VDAC Cell Signaling Technology #4661
Monoclonal Rabbit anti-TGOLN2/TGN38 Cell Signaling Technology #95649
Monoclonal Rabbit anti-CD81 Cell Signaling Technology #10037
Monoclonal Rabbit anti-Rab27A Cell Signaling Technology #69295
Monoclonal Rabbit anti-Rab27B Cell Signaling Technology #17572
Polyclonal Rabbit anti-Rab27B Cell Signaling Technology #44813
Monoclonal Mouse anti-Alix Cell Signaling Technology #2171
Monoclonal Rabbit anti-CD73 Cell Signaling Technology #13160
Monoclonal Rabbit anti-VISTA Cell Signaling Technology #64953
Monoclonal Rabbit anti-PVR Cell Signaling Technology #13544
Monoclonal Rabbit anti-PD-L1 Cell Signaling Technology #15165
Monoclonal Rabbit anti-PD-L1 Cell Signaling Technology #29122
Polyclonal Rabbit anti-CD147 Thermofisher Scientific #34-5600
Polyclonal Rabbit anti-CD47 Thermofisher Scientific #PA5-116827
Monoclonal Rabbit anti-CD155 Thermofisher Scientific #MA5-29762
Monoclonal Mouse anti-β-Actin Sigma-Aldrich A5316
Monoclonal Mouse anti-CD29 BD Transduction Laboratories 610467
Monoclonal Mouse anti-TSG101 BD Transduction Laboratories 612696
Monoclonal Mouse anti-CD63 BD Transduction Laboratories 556019
Monoclonal Mouse anti-Flotillin-1 BD Transduction Laboratories 610820
Monoclonal Rabbit anti-B7-H3/CD276 Bethyl Laboratories #A700-025
Monoclonal Rabbit anti-VISTA Bethyl Laboratories #A700-035
Goat anti-rabbit IgG, HRP-linked Antibody Cell Signaling Technology #7074
Goat anti-mouse 488 Life Technologies A11001
Goat anti-rabbit 488 Life Technologies A11034
Goat anti-mouse 568 Life Technologies A11004
Goat anti-rabbit 568 Life Technologies A11036
Goat anti-mouse 647 Life Technologies A32728

	Goat anti-rabbit 647 Life Technologies A32733<br>Donkey anti-Mouse IgG (H+L) Highly Cross-Adsorbed Secondary Antibody, Alexa Fluor™ Plus 680 Thermo Fisher Scientific A32788<br>Goat anti-Mouse IgG (H+L) Highly Cross-Adsorbed Secondary Antibody, Alexa Fluor™ Plus 800 Thermofisher Scientific A32730<br>Donkey anti-Rabbit IgG (H+L) Highly Cross-Adsorbed Secondary Antibody, Alexa Fluor™ Plus 800 Thermofisher Scientific A32808	
Validation	Each antibody used for Western blotting was validated by the company from which is was purchased for that application. Each antibody used for immunofluorescence was validated by the company from which it was purchased for that application.	

Eukaryotic cell lines

Policy information about cell lines and Sex and Gender in Research

Cell line source(s)	Human DKO-1 (male) colon cancer ordered from ATCC, human Gli36 glioblastoma ordered from ATCC, human MDA-MB-231 (female) breast cancer cells lines ordered from ATCC, human CCD-18Co (female) colon fibroblast cells ordered from ATCC, murine B16-F1 (male) melanoma cells ordered from ATCC, MV3 melanoma cells from ATCC, and mouse embryonic fibroblasts from Dr. Jennifer Lippincott-Schwartz
Authentication	The DKO-1, MDA-MB-231, CCD-18Co, and B16-F1 were authenticated by ATCC. The mouse embryonic fibroblasts were not authenticated.
Mycoplasma contamination	Cells were tested for mycoplasma via a DAPI stain which did not reveal a non eukaryotic cell specific signal.
Commonly misidentified lines (See ICLAC register)	No cells used in this study are on this list.

Animals and other research organisms

Policy information about studies involving animals; ARRIVE guidelines recommended for reporting animal research, and Sex and Gender in Research

Laboratory animals	The zebrafish line LH1066 was used according to institutional ethical guidelines and used from 0-72 hours post fertilization. 8-week-old female C57BL/6J mice were used according to institutional ethical guidelines.
Wild animals	N/A
Reporting on sex	For the bone marrow experiment, all mice used in this study were female. The experiment was repeated three times in which bone marrow was extracted from a single mouse each time. For zebrafish experiments gender was not a variable. Embryos do not have differentiated gonads and have the potential to develop into either ovaries or testes; a process that does not happen until past 10 days post fertilization.
Field-collected samples	N/A
Ethics oversight	All animal studies were done in accordance with NIH, the US Department of Agriculture Animal Welfare Act, and the US Public Health Service Policy on Humane Care and Use of Laboratory Animals and were approved by Vanderbilt University Medical Center's Institutional Animal Care and Use Committee. Bone marrow from mice were collected according to M1800191-01. Zebrafish embryo experiments were conducted in accordance with M2100073-00-S2300172.

Note that full information on the approval of the study protocol must also be provided in the manuscript.
