# Supplementary material for: Blebbisomes are large, organelle-rich extracellular vesicles with cell-like properties
Source: Nat Cell Biol. 2025 Feb 21;27(3):438–48. doi: 10.1038/s41556-025-01621-0 (PMC11906356; doi:10.1038/s41556-025-01621-0)

## Fig 2c

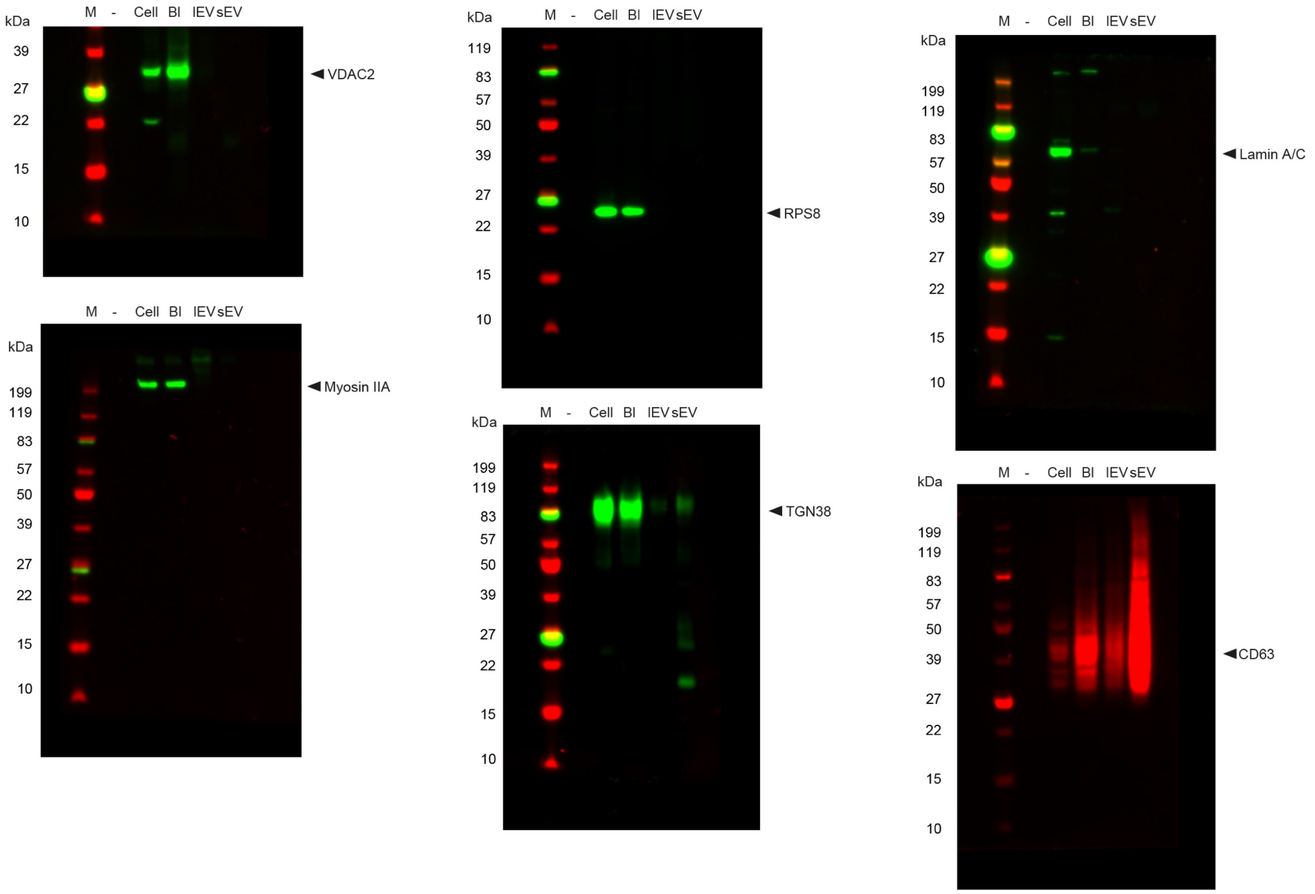
M - Cell Bl lEV sEV
kDa
39 27 22 15 10
VDAC2
199 119 83 57 50 39 27 22 15 10
Myosin IIA
119 83 57 50 39 27 22 15 10
RPS8
199 119 83 57 50 39 27 22 15 10
TGN38
199 119 83 57 50 39 27 22 15 10
Lamin A/C
CD63

## Fig 2d

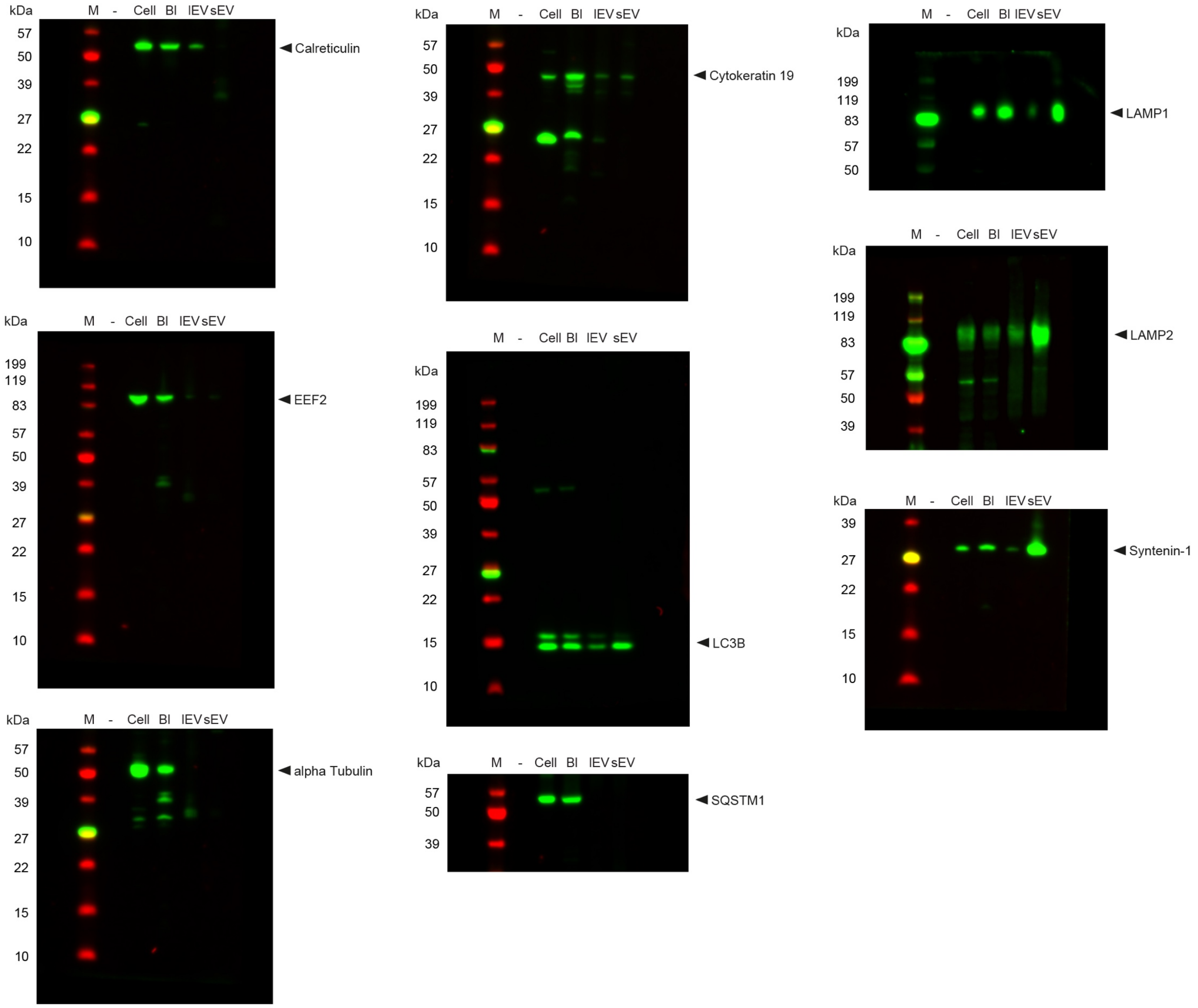
M - Cell Bl lEV sEV
kDa
57 50 39 27 22 15 10
Calreticulin
199 119 83 57 50 39 27 22 15 10
EEF2
57 50 39 27 22 15 10
alpha Tubulin
Cytokeratin 19
LC3B
57 50 39
SQSTM1
199 119 83 57 50
LAMP1
199 119 83 57 50 39
LAMP2
39 27 22 15 10
Syntenin-1

**Fig 6c**

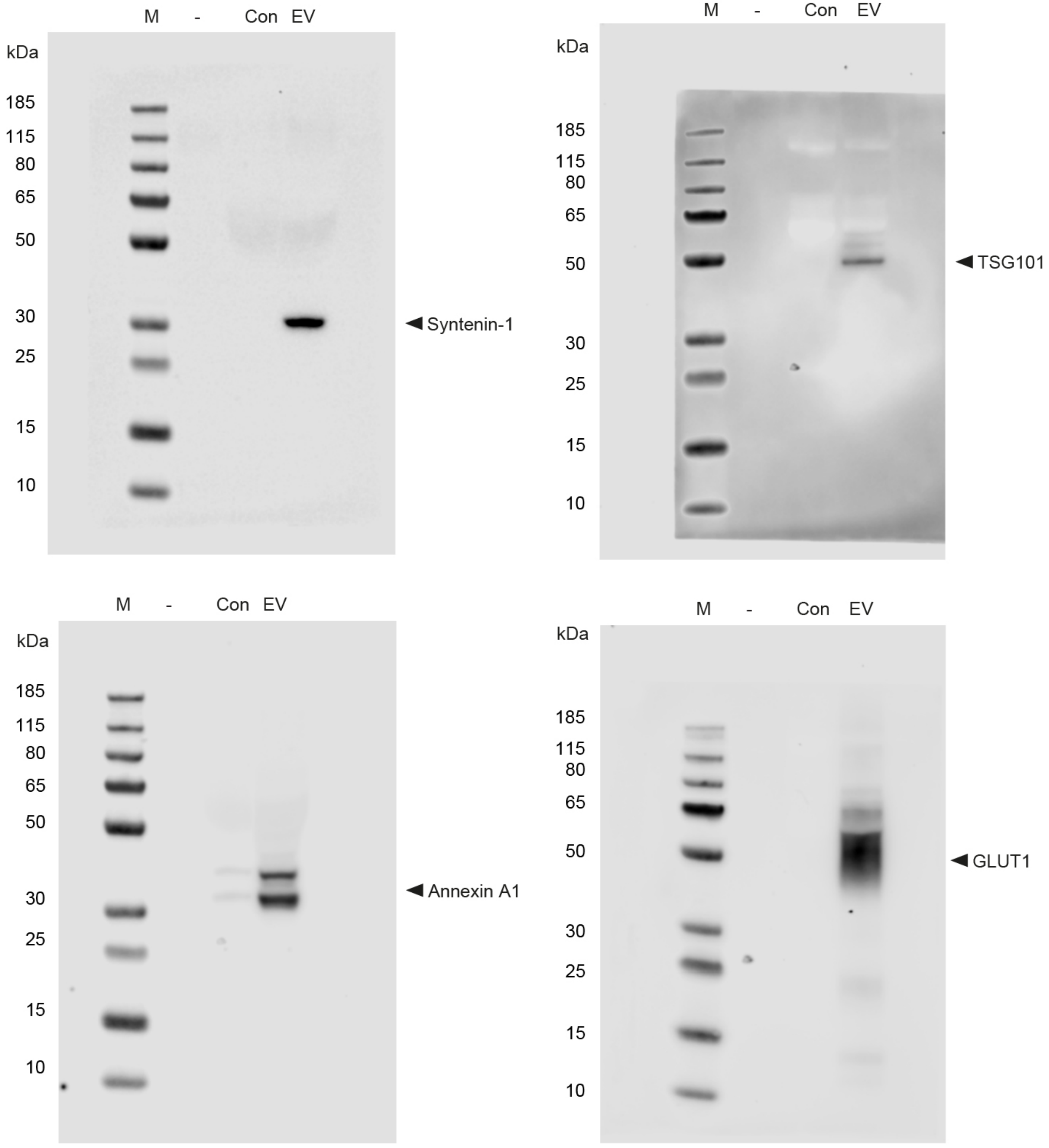
M - Con EV
kDa
185
115
80
65
50
30
25
15
10
◀ Syntenin-1
◀ TSG101
◀ Annexin A1
◀ GLUT1

**Fig 7d**

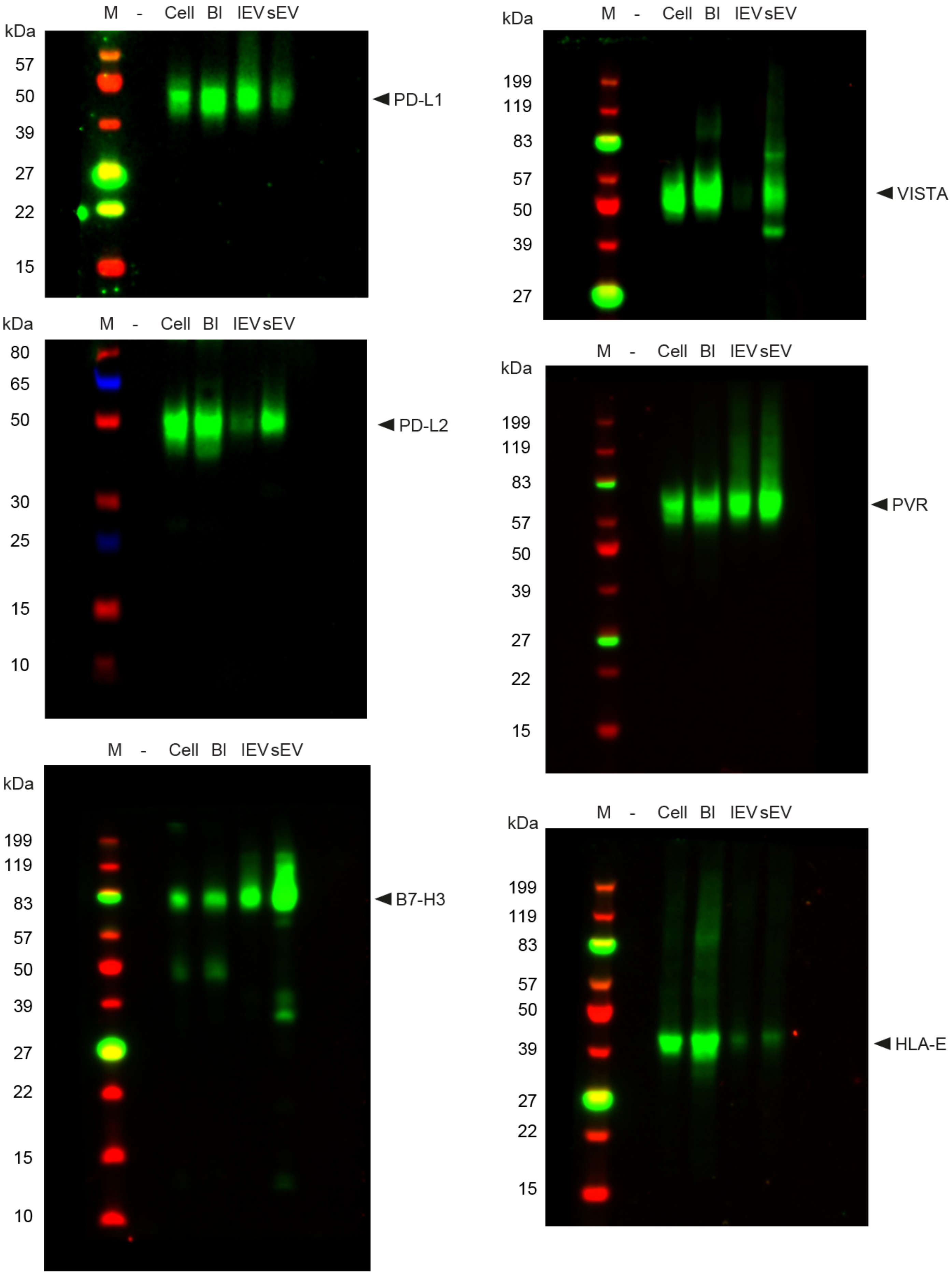

M - Cell Bl lEV sEV
kDa
57
50
39
27
22
15
PD-L1
kDa
M - Cell Bl lEV sEV
199
119
83
57
50
39
27
VISTA
kDa
M - Cell Bl lEV sEV
80
65
50
30
25
15
10
PD-L2
M - Cell Bl lEV sEV
kDa
199
119
83
57
50
39
27
22
15
PVR
M - Cell Bl lEV sEV
kDa
199
119
83
57
50
39
27
22
15
10
B7-H3
M - Cell Bl lEV sEV
kDa
199
119
83
57
50
39
27
22
15
HLA-E

## Extended Data Fig 3b

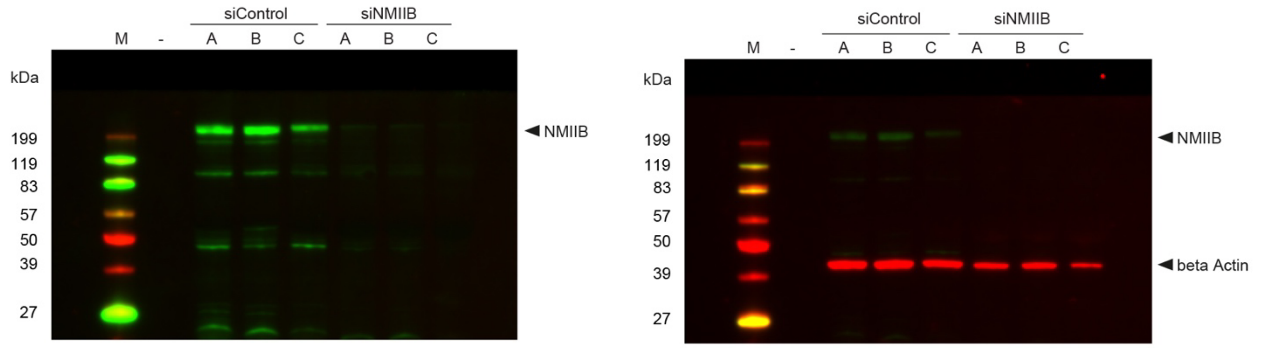


## Extended Data Fig 3c

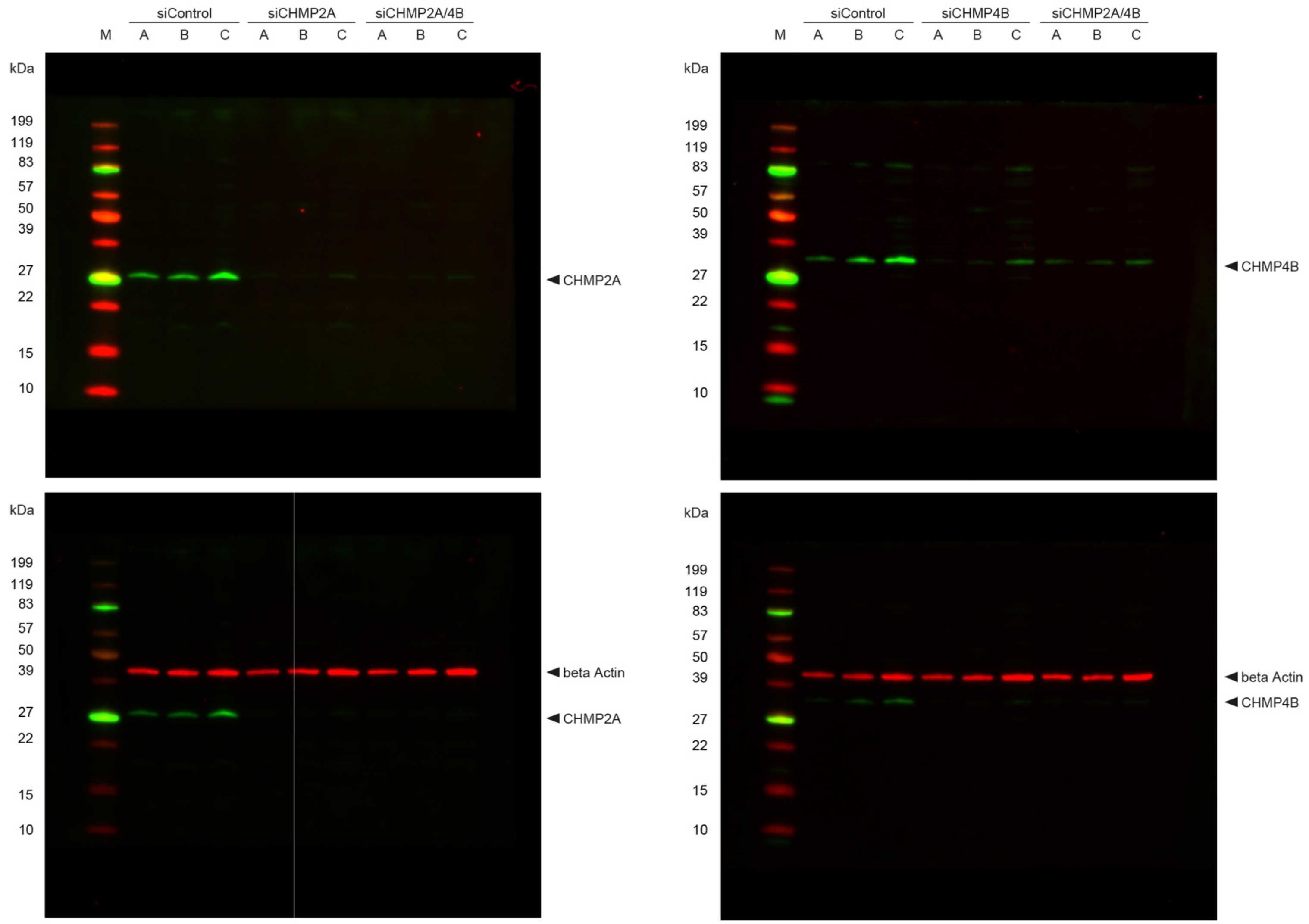

**Extended Data Fig 4b**

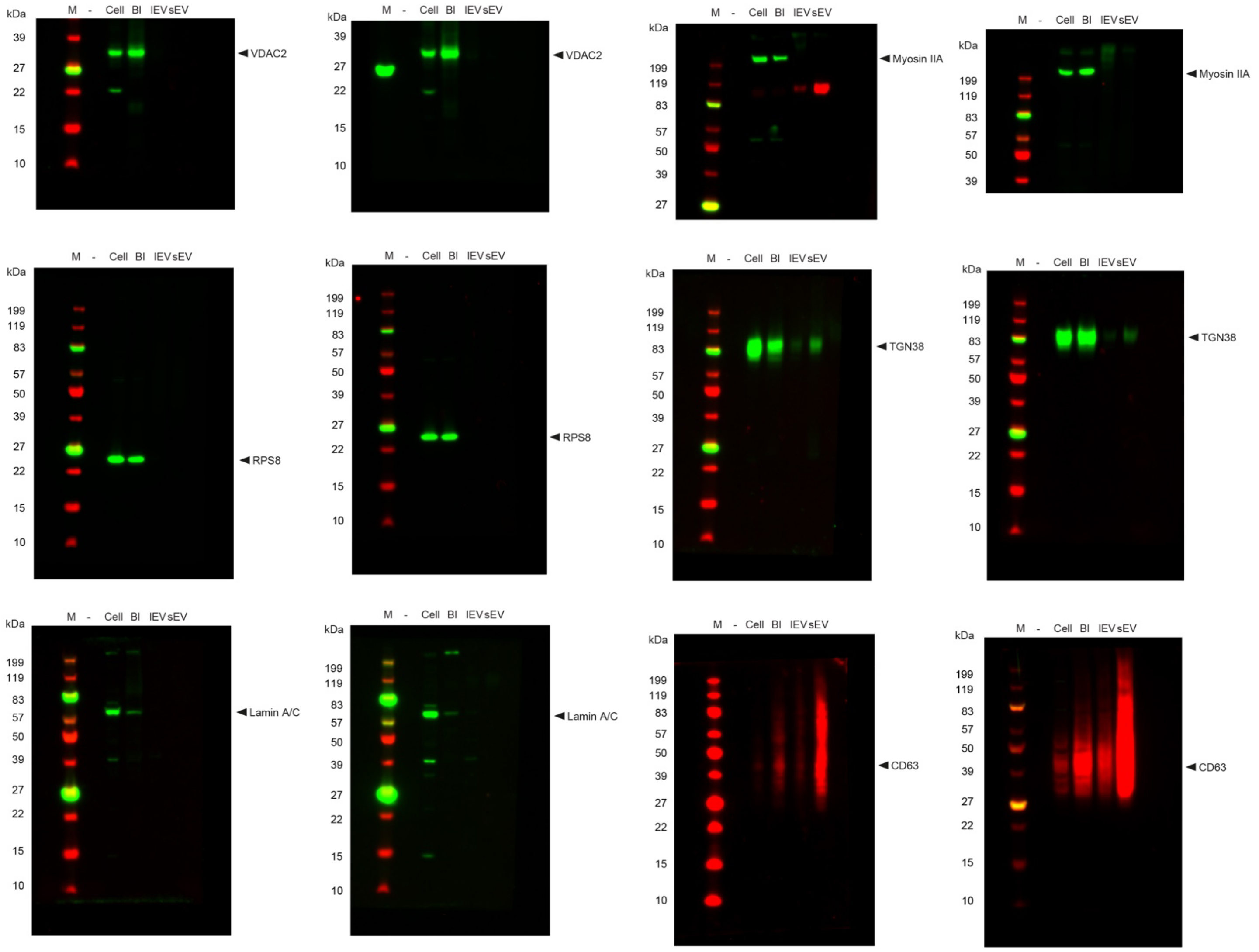

## Extended Data Fig 4c (MDA-MB-231)

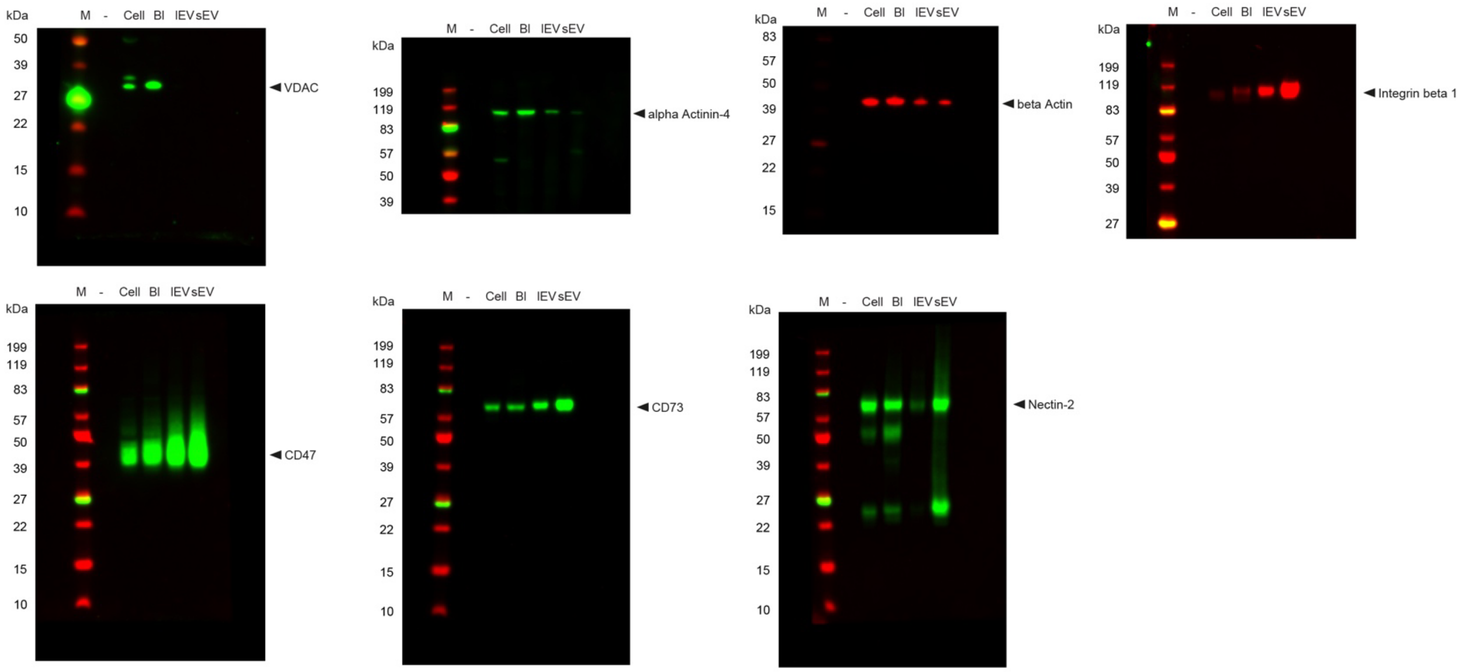


## Extended Data Fig 4c (B16-F1)

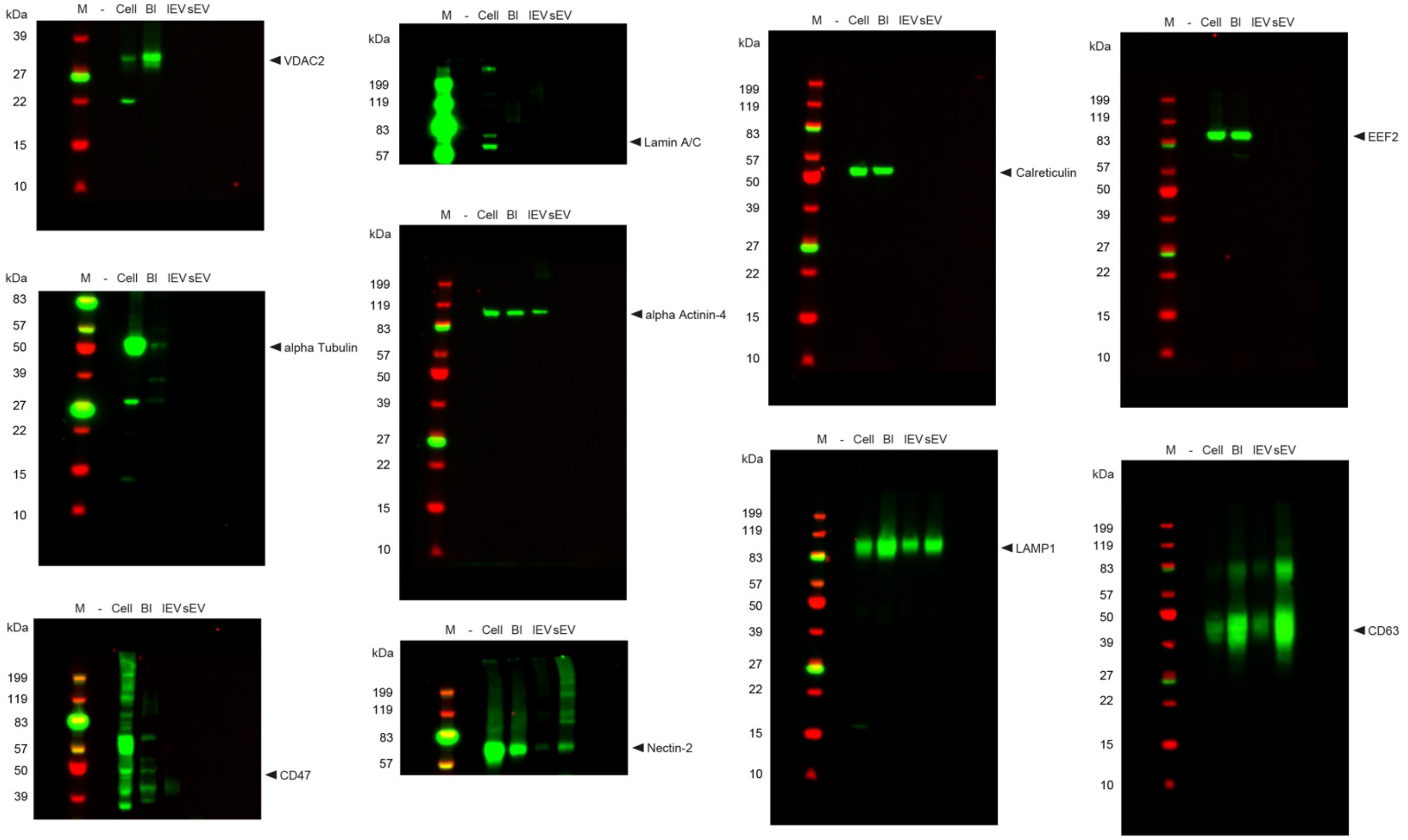

## Extended Data Fig 4c (Gli36)

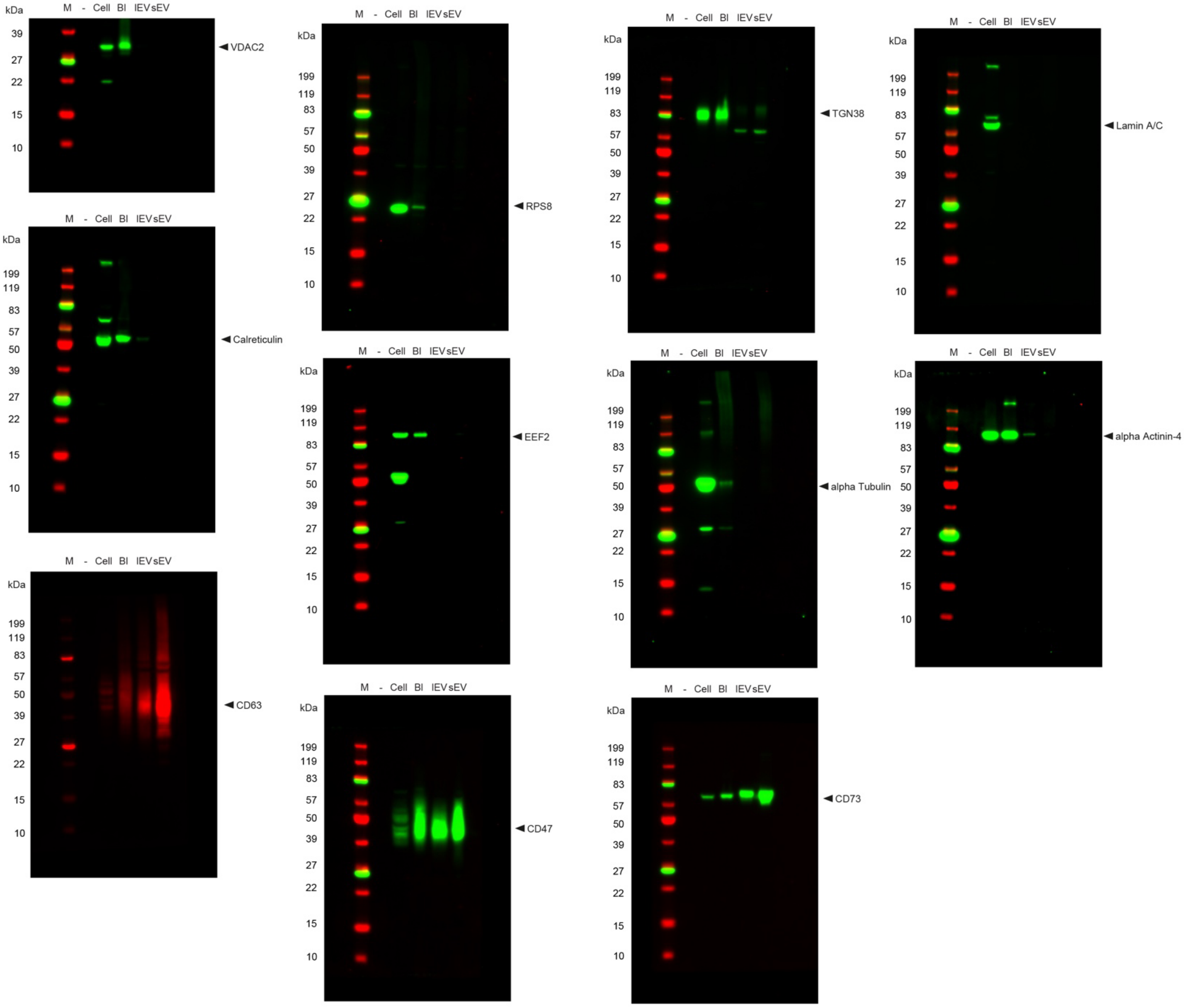

## Extended Data Fig 7b (MDA-MB-231)

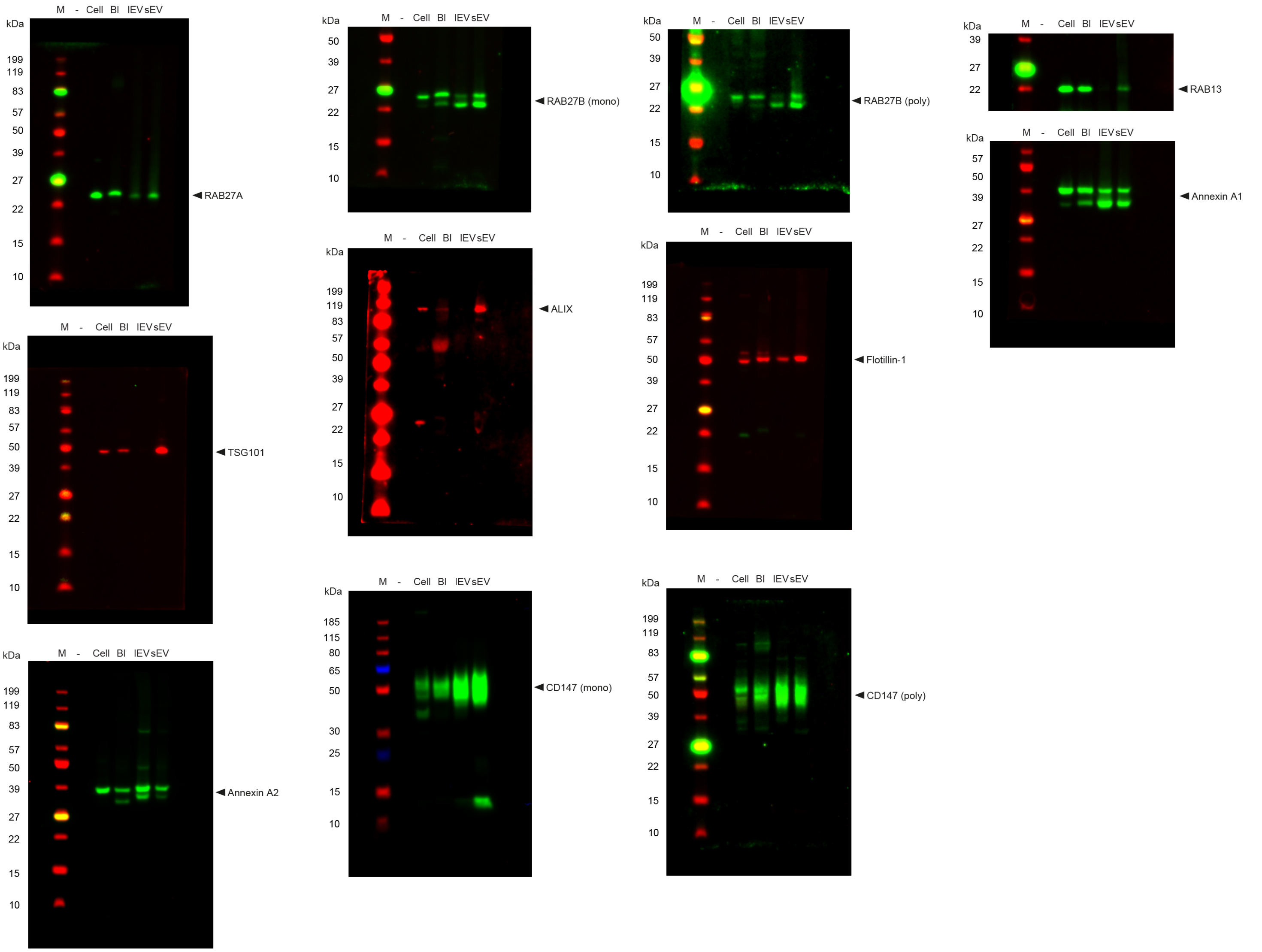


## Extended Data Fig 7b (B16-F1)

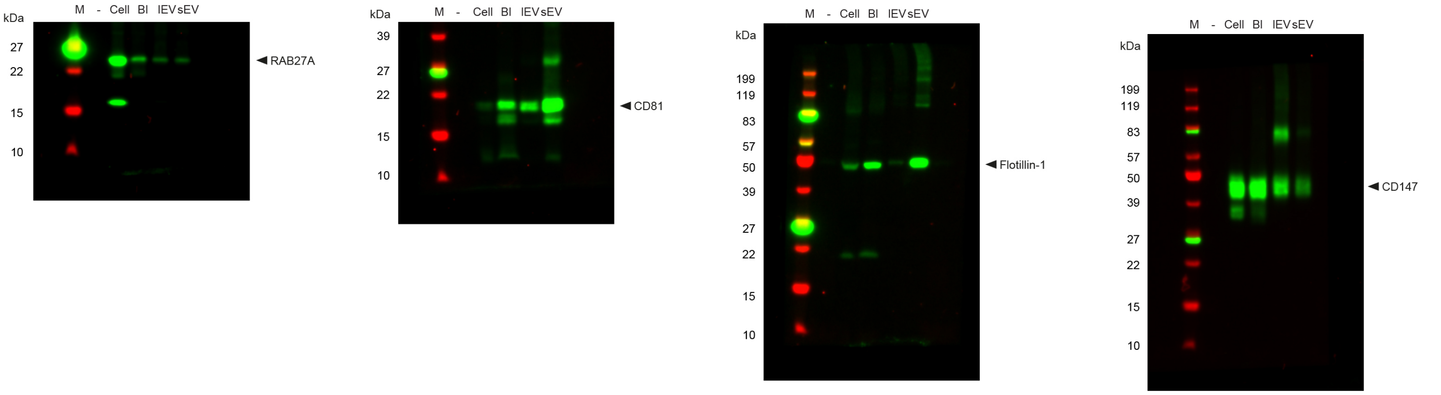

## Extended Data Fig 7c

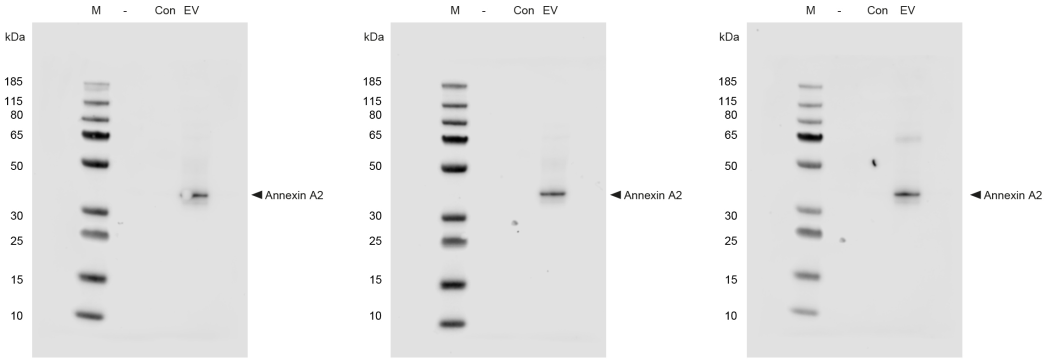


## Extended Data Fig 7d

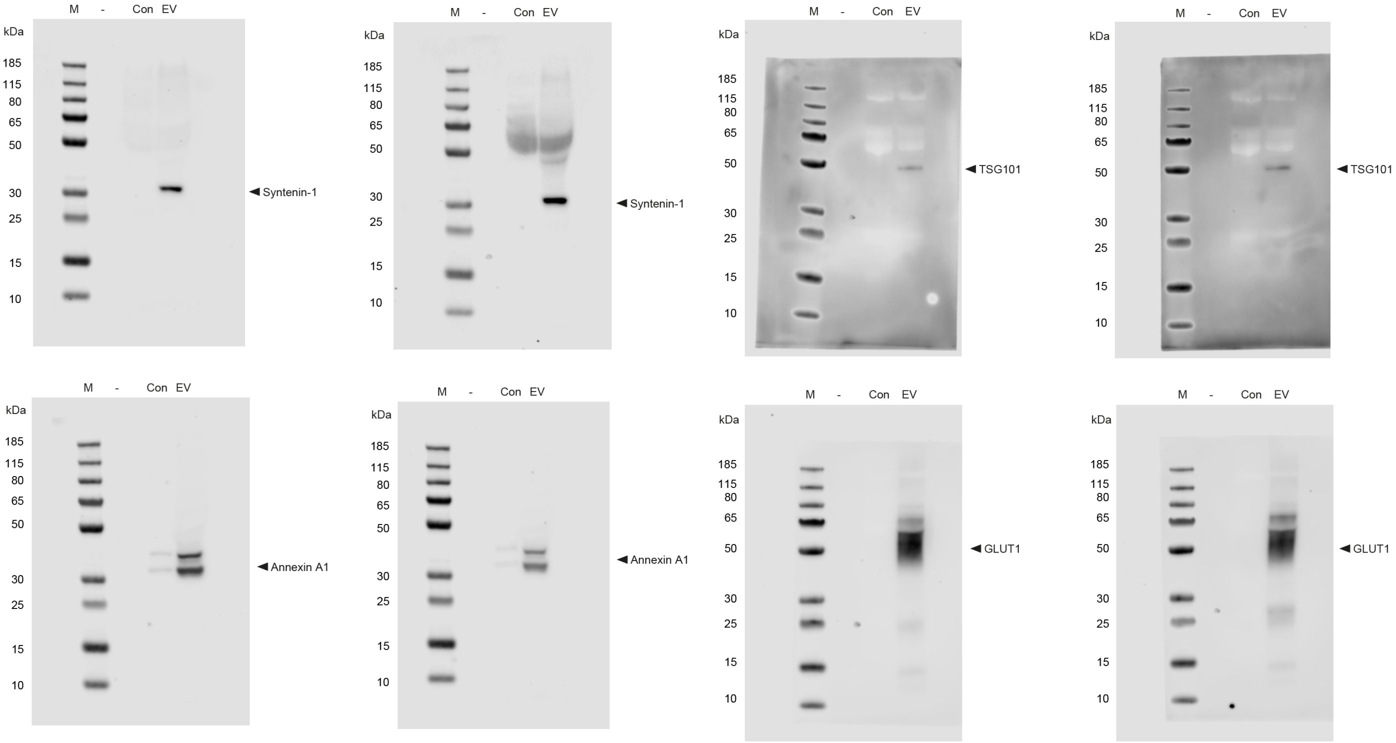

**Extended Data Fig 8a**

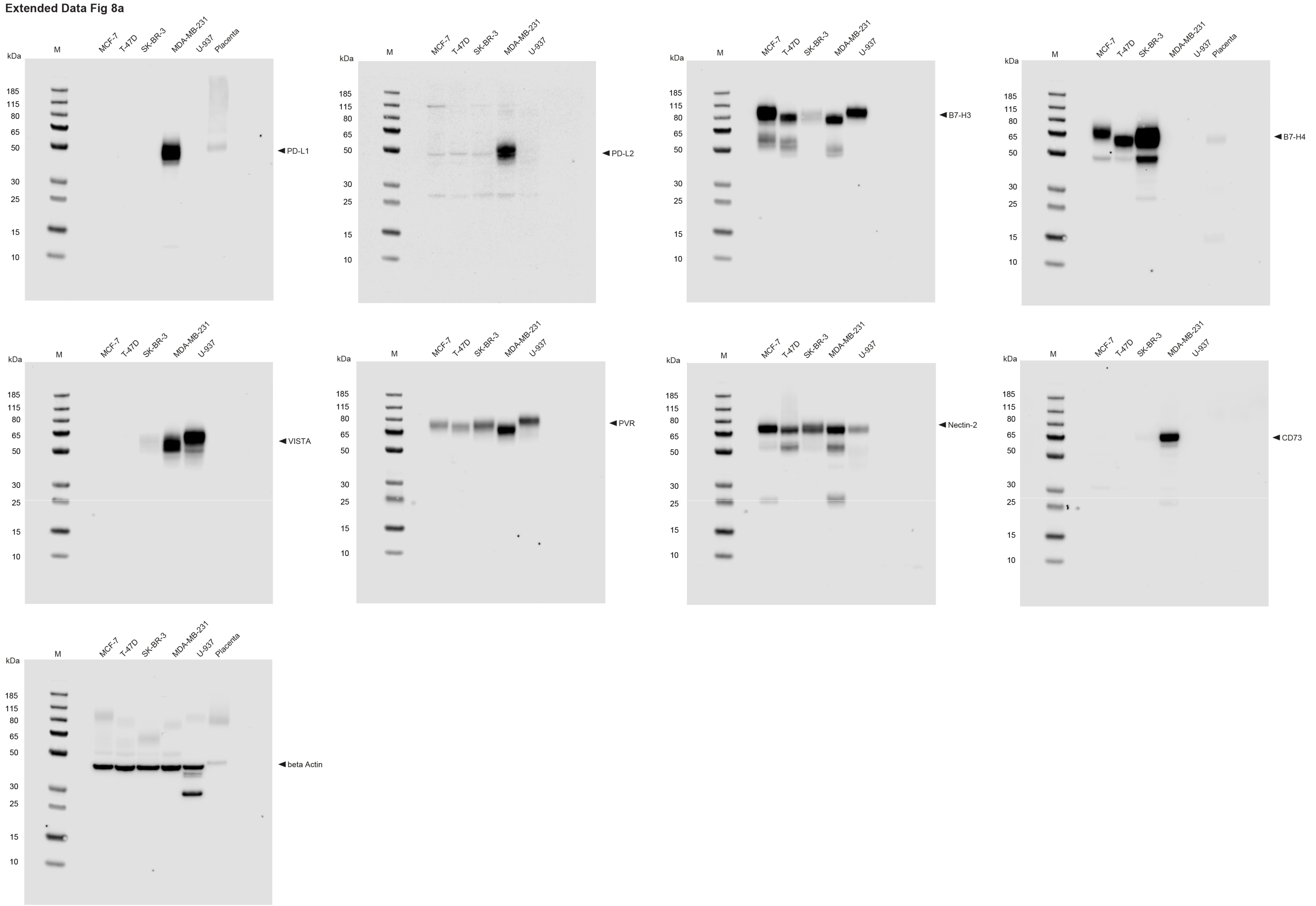


**Extended Data Fig 8b**

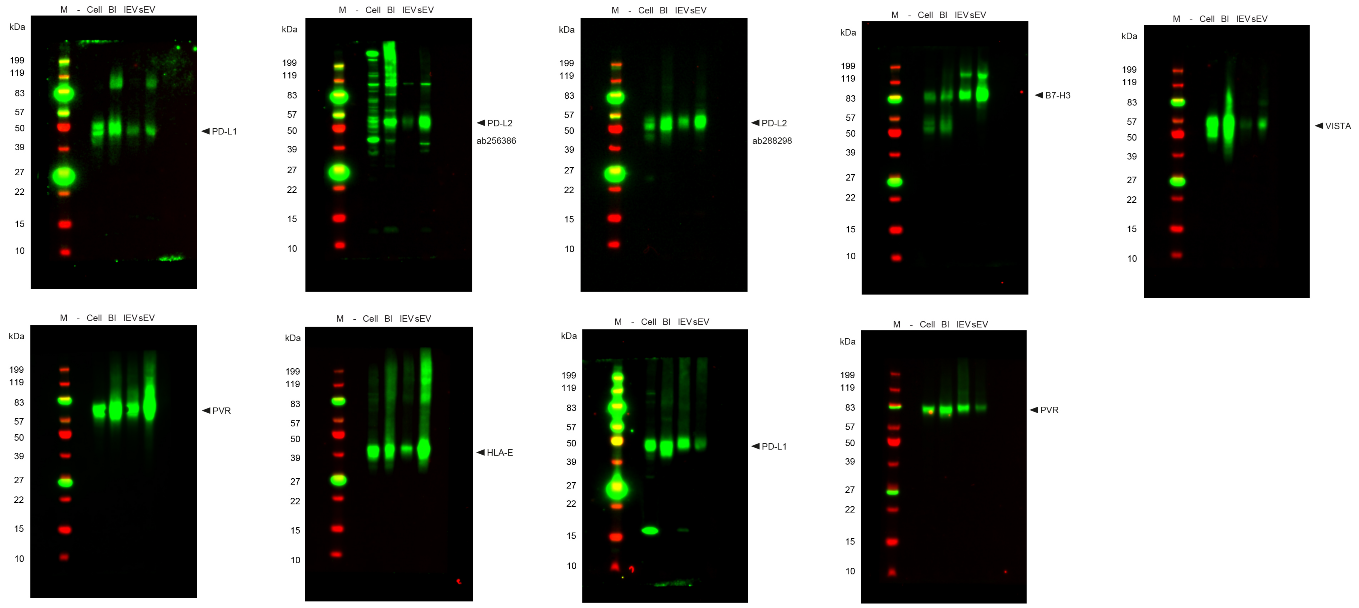

**Extended Data Fig 8c**

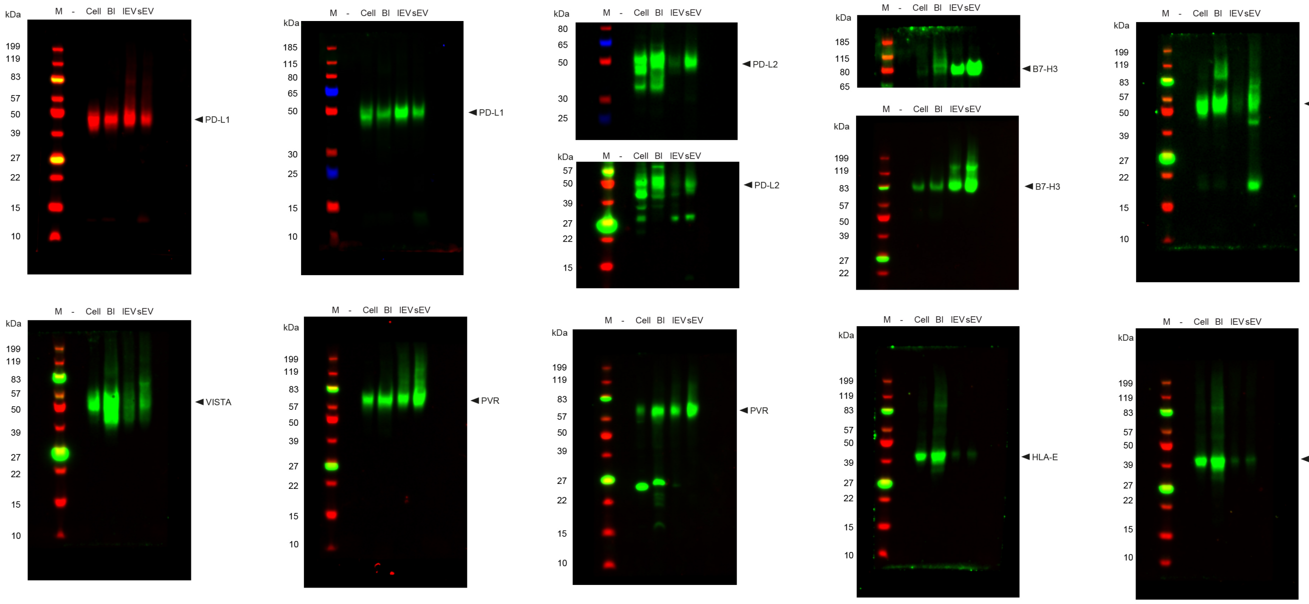


**Extended Data Fig 8d**

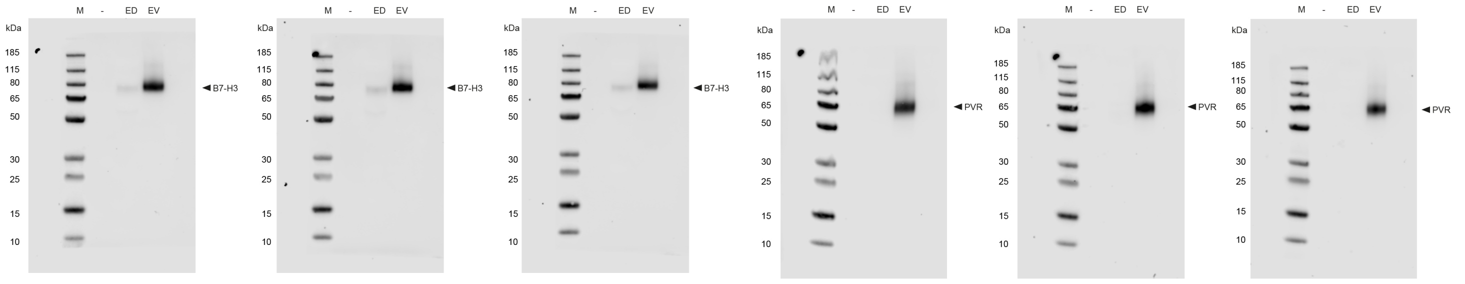

Supplement: Supplementary file 19 — Unprocessed western blots. [file 41556_2025_1621_MOESM19_ESM.pdf]
